# Time Course of Hemostatic Disruptions After Traumatic Brain Injury: A Systematic Review of the Literature

**DOI:** 10.1007/s12028-020-01037-8

**Published:** 2020-06-30

**Authors:** Alexander Fletcher-Sandersjöö, Eric Peter Thelin, Marc Maegele, Mikael Svensson, Bo-Michael Bellander

**Affiliations:** 1 Department of Neurosurgery, Karolinska University Hospital, Stockholm, Sweden; 2 Department of Clinical Neuroscience, Karolinska Institutet, Bioclinicum J5:20, 171 64 Solna, Stockholm, Sweden; 3 Department of Neurology, Karolinska University Hospital, Stockholm, Sweden; 4 Department for Trauma and Orthopedic Surgery, Cologne-Merheim Medical Center, University Witten/Herdecke, Cologne, Germany; 5 Institute for Research in Operative Medicine, University Witten/Herdecke, Cologne, Germany

**Keywords:** Traumatic brain injury, Hemostasis, Coagulation, Coagulopathy, Thrombosis

## Abstract

Almost two-thirds of patients with severe traumatic brain injury (TBI) develop some form of hemostatic disturbance, which contributes to poor outcome. While the initial head injury often leads to impaired clot formation, TBI is also associated with an increased risk of thrombosis. Most likely there is a progression from early bleeding to a later prothrombotic state. In this paper, we systematically review the literature on the time course of hemostatic disruptions following TBI. A MEDLINE search was performed for TBI studies reporting the trajectory of hemostatic assays over time. The search yielded 5,049 articles, of which 4,910 were excluded following duplicate removal as well as title and abstract review. Full-text assessment of the remaining articles yielded 33 studies that were included in the final review. We found that the first hours after TBI are characterized by coagulation cascade dysfunction and hyperfibrinolysis, both of which likely contribute to lesion progression. This is then followed by platelet dysfunction and decreased platelet count, the clinical implication of which remains unclear. Later, a poorly defined prothrombotic state emerges, partly due to fibrinolysis shutdown and hyperactive platelets. In the clinical setting, early administration of the antifibrinolytic agent tranexamic acid has proved effective in reducing head-injury-related mortality in a subgroup of TBI patients. Further studies evaluating the time course of hemostatic disruptions after TBI are warranted in order to identify windows of opportunity for potential treatment options.

**Electronic supplementary material:**

The online version of this article (10.1007/s12028-020-01037-8) contains supplementary material, which is available to authorized users.

## Background

Despite improvements in medical triage and tertiary care, traumatic brain injury (TBI) is associated with significant morbidity and mortality [1]. The initial injury is often followed by hemostatic disturbance, which is present in up to two-thirds of patients with severe TBI and associated with increased mortality [2, 3]. While it is still unclear exactly how TBI affects the coagulation system, the primary drivers appear to be platelet dysfunction, endothelial activation, disturbed fibrinolysis, endogenous anticoagulation, and inflammation [2, 4–6].

There is controversy regarding the exact nature of hemostatic disruption after TBI, and evidence exists to support the presence of both a hyper- and hypocoagulable state [2]. While the initial head injury often leads to impaired clot formation and exacerbation of hemorrhagic lesions [2, 7–9], TBI is also independently associated with an increased risk of venous thromboembolism [10–13] and ischemic stroke [14–18]. Most likely, there is a progression from early increased bleeding risk to a later prothrombotic state. However, an overlap and lack of distinction between the phases exists.

In this paper, we systematically review the literature on the time course of hemostatic disruptions following TBI, as reflected by temporal changes in selected laboratory assays, in order to highlight current evidence and potential future directions.

## Methods

### Search Strategy and Selection Criteria

A search strategy was decided upon (Supplementary file 1) and carried out using the MEDLINE database from its date of inception until December 2019. Titles and abstracts were independently screened to determine whether they met the inclusion criteria. Full texts of the chosen articles were assessed to confirm this. Reference lists of relevant articles were screened for additional studies.

### Inclusion and Exclusion Criteria

Studies of adult and pediatric TBI patients that reported the time course (> 1 sample per patient) of one or several hemostatic assays were eligible for inclusion. The assays of primary interest were platelet count, prothrombin time (PT), partial thromboplastin time (PTT), activated partial thromboplastin time (APTT), D-dimer, thrombin antithrombin III complex (TAT), Prothrombin fragment 1 + 2 (F1 + 2), tissue plasminogen activator (t-PA), plasminogen activator inhibitor-1 (PAI-1), thromboelastography (TEG), thromboelastometry (TEM), Multiplate® (Roche Diagnostics, Basel, Switzerland) and VerifyNow® (Accumetrics, San Diego, CA, USA). There were no restrictions on methodological quality. Studies were excluded if they were non-English, and if it wasn’t possible to interpret data relating to patients with TBI (e.g. multitrauma studies without a subgroup analysis) or a specific laboratory assay (e.g. studies that grouped several assays together as one). For studies evaluating the effect of a hemostatic intervention, only data for the control group were extracted.

### Data Abstraction

Using a customized form, we extracted data from the included studies and stored them in an electronic database. Where applicable, the following data were abstracted: study design, patient ages, cohort size, TBI severity, presence of extracranial injuries, pre-injury antithrombotic or anticoagulation medication, sampling times, time from injury to first sample, and the temporal trajectory for each assay. No meta-analysis was possible due to the heterogeneity of the data.

## Results

### Study Selection and Characteristics

**Fig. 1:**
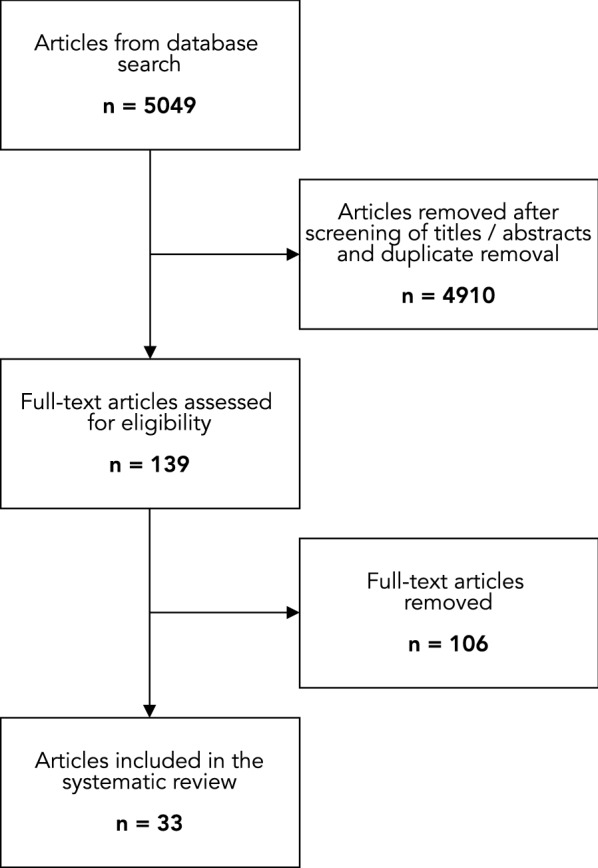
Schematic overview of the number of identified records for the systematic steps of the review process. Abbreviations: APTT = activated partial thromboplastin time; PT = prothrombin time; PTT = partial thromboplastin time

The initial literature search generated 5,049 articles, of which 4,910 were excluded following duplicate removal as well as title and abstract review. Full-text assessment of the remaining 139 articles yielded 33 studies that were included in the final review (Fig. 1).

### Platelets

#### Platelet Count

We included 17 studies with platelet count data, of which all but one found that platelet count decreased after injury [19–34]. In the largest study of 274 adults with severe TBI, mean platelet count was normal on admission and then decreased over time, with a steeper decline in more severe cases [20]. A similar pattern of posttraumatic decrease in platelet count was seen in the prospective studies [22, 23, 28] as well as in mild TBI [32]. Most often the platelet counts remained within reference ranges [19, 20, 24–26, 29, 30, 35], but some studies reported on the development of posttraumatic thrombocytopenia as well [21, 27, 28, 32]. The lowest platelet count levels were seen 1–5 days after injury [27–32], followed by a rebound to admission levels by day 5–9 [28, 30, 31]. One study found no change in platelet count over time, but only sampled blood tests on admission and 10–17 days after injury [35] (Table 1).

**Table 1:** Platelet count

Reference	Study design	TBI type	Age category	Patients (n)	Hemostatic comorbidities	Sampling times	First sample	Result
Nakae [19, 20]	Retrospective	Isolated severe TBI	Adults	234 [18], 274 [19]	Excluded those with liver failure, hematological disease or malignancy	On admission and 3, 6, 12 h after injury	< 1 h after injury	Decrease in PLT count during time studied. Faster decrease in more severe cases. Median values stayed within reference range
Grenander [21]	RCT	Isolated TBI	Adults	28	Excluded those with known bleeding or coagulation disorders	On admission and 8, 16, 24, 36, 48, 72, 96, 120 h after injury	Mean 8.6 h after injury	Decrease in PLT count between admission and 48 h after injury, reaching an average level of 120 × 10^9^/L, followed by an increase that did not reach normal values during time studied
Bredbacka [22]	Prospective observational	Isolated TBI	Adults	20	N/A	On admission and 1, 2, 3, 4, 5, 6 days after injury	< 24 h of injury	Decrease in PLT count that reached its lowest point 2 days after injury, with 50% of patients showing values < 150 × 10^9^/L, followed by an increase that reached admission-values 5 days after injury
Kutcher [23]	Prospective observational	TBI	N/A	62	N/A	On admission and 6, 12, 24, 48, 72, 96, 120 h after injury	N/A	Decrease in PLT count between admission and 48 h after injury, followed by an increase that did not reach admission-values within time studied. Mean platelet count remained above 100 × 10^9^/L
Vecht [24]	Prospective observational	Moderate to severe TBI	N/A	34	N/A	On admission and 1, 2, 3, 4, 5, 9, 16 days after injury	N/A	Decrease in PLT count between admission and 2–5 days after injury, followed by an increase that reached admission-values at 9 days after injury. Values within reference range
Auer [25]	Prospective observational	Severe isolated TBI	N/A	30	N/A	On admission and during the first 10 days after injury	N/A	Decrease in PLT count between admission and 3–5 days after injury, followed by an increase that reached admission-values by end of first week. Unclear if within reference range
Carrick [26]	Retrospective	Isolated moderate to severe TBI	All ages	184	N/A	On admission and 72 h after injury	N/A	Thrombocytopenia in 14% on admission that increased to 46% after 72 h
Hulka [27]	Retrospective	TBI	Adults	91	N/A	N/A	3.1 ± 0.4 h	Thrombocytopenia in 0% on admission and in 30% 2–6 h after injury, followed by a decrease to 20% after 18–24 h
Engström [28]	Retrospective	Severe TBI	N/A	27	N/A	On admission and 24 h after injury	N/A	Decrease in PLT count over time. Some patients were below reference range but mean value unclear
Lustenberger [29]	Retrospective	Isolated severe TBI	N/A	278	N/A	On admission and every 6 h until clinically stable	N/A	Decrease in PLT count between admission and 24 h after injury, followed by an increase that did not reach admission values during time studied. Mean value below reference range at lowest point
Nekludov [30]	Prospective observational	Isolated severe TBI	Adults	20	Alcoholism in 50%. Excluded those with other known coagulation disorders	On admission and 3 days after injury	17 ± 12 h after injury	Decrease in PLT count over time. Unclear if within reference range
Fair [31]	Prospective observational	Isolated TBI	Adults	141	Excluded those with known coagulation disorders	On admission and 6, 12, 24, 48 h after injury	N/A	Decrease in PLT count over time. Unclear if within reference range
Briggs [32]	Interventional	Isolated TBI	Adults	5	Excluded those with history of thrombocytopenia, platelet disorder, or other preexisting coagulopathy	On admission and 4–12 h after injury	N/A	Decrease in PLT count over time. Values within reference range
Wang [33]	Interventional	Isolated moderate TBI	Adults	83	Excluded those with known coagulation disorders	12, 24, 48, 72 h after injury	N/A	Decrease in PLT count over time. Values within reference range
Scherer [34]	Prospective observational	Isolated severe TBI	Adults	20	N/A	On admission and 3 h after admission	N/A	Decrease in PLT count over time. Values within reference range
Touho [35]	Prospective observational	Isolated TBI	N/A	12	N/A	On admission and 10–17 days after injury	< 24 h of injury	No change in PLT count over time. Values within reference range

*h* hours; *N/A* not available; *RCT* randomized control trial; *TBI* traumatic brain injury

#### Platelet Function

We included 6 studies with platelet function data. The methods used were TEG, Multiplate® (Roche Diagnostics, Basel, Switzerland) and spectrophotometry. All studies found that platelet function decreased after injury [22, 24, 29, 36–38]. This appeared to be independent of platelet transfusion and pre-injury antithrombic therapy [36, 37]. In the largest prospective study of 153 adults with TBI, mean adenosine diphosphate (ADP)-induced aggregation was below-normal on admission and continued to decrease during the first 24 h after injury [36]. Five other studies reported a similar pattern of normal to below-normal platelet function on admission that then decreased over time in stimulation to collagen (COL) [24], ADP, thrombin receptor activating peptide (TRAP) and arachidonic acid (AA or ASPI) [22, 37]. The lowest platelet function levels were observed 6–48 h after injury [24, 29, 37, 38] and then returned to normal after 2–16 days [22, 29, 37, 38]. In the two studies that observed patients for more than one week, platelet function increased to above-normal levels (i.e. hyperactive platelets) following their initial decline [29, 37] (Table 2).

**Table 2:** Platelet function

Reference	Study design	TBI type	Age category	Patients (n)	Antithrombotic treatment (n, %)	Hemostatic comorbidities	Sampling times	First sample	Result
Nekludov [30]	Prospective observational	Isolated severe TBI	Adults	20	0 (0%)	Alcoholism in 50%. Excluded those with other known coagulation disorders	On admission and 3 days after injury	17 ± 12 h after injury	Below-normal platelet function on admission (Multiplate: AA/ASPI and ADP), that returned to normal by day 3 in most cases although some patients normalized after 2–3 weeks
Vecht [38]	Prospective observational	Moderate to severe TBI	N/A	34	N/A	N/A	On admission and 1, 2, 3, 4, 5, 9 days and 2, 3, 4, 5, 6 weeks after injury	< 2 h of injury	Below-normal platelet function on admission (Vitatron spectrophotometer: ADP), with the lowest value on day 2, that returned to normal after 9–16 days
Kutcher [23]	Prospective observational	TBI	N/A	62	5 (8%)	N/A	On admission and 6, 12, 24, 48, 72, 96, 120 h after injury	N/A	Low-to-normal platelet function on admission (Multiplate: ADP, TRAP, AA/ASPI, COL), that decreased to below-normal levels 6 h after injury, before returning to normal after 24 h (TRAP, COL), 96 h (ADP) or 120 h (AA/ASPI)
Briggs [32]	Prospective interventional	Isolated TBI	Adults	5	0 (0%)	Excluded those with history of thrombocytopenia, platelet disorder, or other preexisting coagulopathy	On admission and 4–12 h after injury	N/A	Below-normal platelet function on admission (Multiplate: COL), with the lowest value after 4–12 h. Same trend for AA/ASPI but within reference ranges
Lindblad [37]	Retrospective	TBI, admitted to ICU	Adults	178	47 (26%)	N/A	N/A	N/A	﻿Below-normal and decreasing platelet function (Multiplate®: AA/ASPI) during first 10 h after injury, that returned to normal after 4–5 days. Results independent of COX-inhibition and platelet transfusion
Guillotte [36]	Prospective observational	TBI	Adults	153	62 (41%)	N/A	N/A	N/A	Below-normal and decreasing platelet function in moderate-to-severe TBI (TEG: ADP) during first 24 h after injury. Results independent of antithrombotic medication

*AA/ASPI* acetylsalicylic acid test; *ADP* adenosine diphosphate test; *COL* collage test; *h* hours; *N/A* not available; *TBI* traumatic brain injury; *TEG* thromboelastography; *TRAP* thrombin receptor activating peptide-6 test

### Coagulation Cascade

#### PT, PTT and APTT

We included 12 studies with PT, PTT and/or APTT data. Most of these studies noted that TBI was followed by a decrease in markers of coagulation cascade function [19, 21, 32, 33, 39, 40]. In the largest study of 441 patients with isolated TBI, mean APTT was increased during the first 12 h after injury, with a mean value of 16 s (reference range: 10.3–13.4 s) [39]. A similar pattern, although within reference ranges, was seen in another study of 234 adult severe TBI patients, where APTT and INR significantly increased during the first 3 h after injury, reaching median values of 29.2 s (reference range: 24–36 s) and 1.07 (reference range: 0.8–1.2), respectively [19]. These observations were further supported by three smaller studies that all identified posttraumatic abnormally increased PT, INR and PTT that peaked at 2–6 h after injury [21, 33, 40]. In addition, one study found that Glasgow Coma Scale (GCS) was inversely proportional to PT, indicating that severer TBI cases induce a more pronounced coagulation cascade dysfunction [40]. Following the initial phase, values then seemed to return to normal after 12 h to several days following injury [32, 33, 40]. Two studies saw no significant change in these coagulation cascade assays over time [23, 25] (Table 3).

**Table 3:** APTT, PT, INR

Reference	Study design	TBI type	Age category	Patients (n)	Antithrombotic treatment (n, %)	Hemostatic comorbidities	Sampling times	First sample	Results
Halpern [39]	Retrospective	Isolated TBI	N/A	441	N/A	N/A	On admission and 6, 12, 18, 24, 48, 72 h after injury	< 1 h of injury	Abnormally elevated APTT during first 12 h after injury, with values then returning to normal
Nakae [19]	Retrospective	Isolated severe TBI	Adults	234	0 (0%)	Excluded those with liver failure, hematological disease or malignancy	On admission and 3, 6, 12 h after injury	< 1 h of injury	Increase in INR and APTT from admission to 3 h after injury. Median values within reference range, with some outliers
Hulka [27]	Retrospective observational	TBI	Adults	91	N/A	N/A	N/A	3.1 ± 0.4 h	Abnormally elevated PT (> 15 s) in 25% on admission that increased to 60% 2–6 h after injury, and then returned to 25% 12–18 h after injury. Also abnormally elevated PTT (> 35 s) in 20% on admission that increased to 60% 2–6 h after injury and then returned to 20% 6–12 h after injury
Touho [35]	Prospective observational	Isolated TBI	N/A	12	N/A	N/A	On admission and 10–17 days after injury	< 24 h of injury	No change in PT or PTT during time studied
Fair [31]	Prospective observational	Isolated TBI	Adults	141	20 (14%) ASA	Excluded those with known coagulation disorders	On admission and 6, 12, 24, 48 h after injury	N/A	INR and APTT showed a trend toward prolongation over time, but stayed within reference ranges
Vecht [24]	Prospective observational	Moderate to severe TBI	N/A	34	N/A	N/A	On admission and 1, 2, 3, 4, 5, 9, 16 days after injury	N/A	PPT decreased on admission and then increased on days 1, 2 and 3 after injury
Carrick [26]	Retrospective observational	Isolated moderate to severe TBI	All ages	184	N/A	N/A	On admission and 72 h after injury	N/A	Increased bleeding (PT > 14.2 s or PTT > 38.4 s) in 21% of patients on admission that increased to 41% 72 h after injury
Pahatouridis [40]	Prospective observational	Isolated moderate TBI	Adults	61	0 (0%)	Excluded those with known coagulopathy	On admission and 4, 6, 24, 48, 72 h after injury	N/A	Abnormally elevated PT in 78% on admission that increased to 100% 3–6 h after injury, before returning to 77% and 36% after 48 and 72 h, respectively. Lower GCS associated with increased PT
Wang [33]	Interventional	Isolated moderate TBI	Adults	83	0 (0%)	Excluded those with known coagulation disorders	12, 24, 48, 72 h after injury	N/A	No change in PT or APTT during time studied
Scherer [34]	Prospective observational	Isolated severe TBI	Adults	20	N/A	N/A	On admission and after 3 h	N/A	Slight increase in APTT over time (within reference ranges), but PT unchanged
Lustenberger [29]	Retrospective	Isolated severe TBI	N/A	278	N/A	N/A	On admission and every 6 h until clinically stable	N/A	Increase in INR from admission to 6 h after injury, which at that point was abnormally elevated. Returned to normal after 60 h

*APTT* activated partial thromboplastin time; *ASA* acetylsalicylic acid; *h* hours; *INR* international normalized ratio; *N/A* not available; *PT* prothrombin time; *PPT* partial thromboplastin time; *TBI* traumatic brain injury

#### TAT and F1 + 2

We included 5 studies with TAT and/or F1 + 2 data. All of these studies found that TAT and F1 + 2 were elevated during the first day of injury [26, 27, 41–43]. In the largest study, which included 28 patients with isolated TBI, mean TAT was elevated on admission (mean 8.6 h after injury) and then decreased to reference levels after 5 days [27]. The other studies reported a similar pattern of increased TAT and/or F1 + 2 levels on admission that then decreased, reaching reference ranges from 24 h up to one week after injury [26, 41–43] (Table 4).

**Table 4:** Thrombin generation: TAT and F1 + 2

Reference	Study design	TBI type	Age category	Patients (n)	Antithrombotic treatment (n, %)	Hemostatic comorbidities	Sampling times	First sample	Results
Grenander [21]	RCT	Isolated TBI	Adults	28	0 (0%)	Excluded those with known bleeding or coagulation disorders	On admission and 8, 16, 24, 36, 48, 72, 96, 120 h after injury	Mean 8.6 h after injury	Elevated TAT on admission that then decreased and reached reference levels 5 days after injury
Nekludov [41]	Prospective observational	Isolated severe TBI	Adults	11	0 (0%)	Excluded those with known coagulation disorders or alcohol abuse	On admission and 3 days after injury	15.4 ± 2.6 h after injury	Elevated TAT and F1 + 2 on admission that decreased and reached reference levels 24 h after injury
Sørensen [42]	Prospective observational	Isolated severe TBI	All ages	14	N/A	N/A	On admission and 1, 2, 3, 7 days after injury	N/A	Elevated TAT and F1 + 2 on admission that then decreased but remained elevated during time studied
Gando [43]	Prospective observational	Isolated TBI	Adults	5	0 (0%)	Excluded those with preexisting coagulation disorders	On admission and 1, 2, 3, 4 days after injury	N/A	Elevated TAT and F1 + 2 on admission that then decreased but remained elevated during time studied
Scherer [34]	Prospective observational	Isolated severe TBI	Adults	20	N/A	N/A	A and 3 h after admission	N/A	Elevated TAT and F1 + 2 on admission that then decreased but remained elevated during time studied

F1 + 2 = prothrombin fragment F1 + 2; *h* hours; *TAT* thrombin–antithrombin complex; *TBI* traumatic brain injury

### Fibrinolysis

#### D-dimer

We included 11 studies with D-dimer data, all of which reported increased levels after injury [19, 23, 26, 27, 33, 35, 40, 41, 43–47]. In the largest prospective study of 141 patients with isolated TBI, mean D-dimer was increased on admission with mean peak levels of 8 µg/mL (normal range: 0.0–1.0 µg/mL) observed 6 h after trauma. This was then followed by a decrease over time [23]. Another study of 234 TBI patients identified a D-dimer peak within 3 h of injury in patients with head abbreviated injury scale (AIS) 5, and within 3–6 h in those with head AIS 4 [19], where median levels reached as high as 45.3 µg/mL. In these studies, D-dimer levels were also correlated with injury severity [19, 40] and hemorrhage progression [23]. A similar pattern of posttraumatic increase in D-dimer was seen in several other smaller studies as well [26, 27, 33, 35, 40, 41, 43–47]. In respect of time to normalization, most studies were limited by short follow-up time [19, 23, 26, 33, 40, 41, 46, 47], but one reported that 55% of patients normalized their D-dimer levels within 3 days of injury [40] (Table 5).

**Table 5:** D-dimer

Reference	Study design	TBI type	Age category	Patients (n)	Antithrombotic treatment (n, %)	Hemostatic comorbidities	Sampling times	First sample	Results
Fair [31]	Prospective observational	Isolated TBI	Adults	141	20 (14%) ASA	Excluded those with known coagulation disorders	On admission and 6, 12, 24, 48 h after injury	N/A	Increased D-dimer on admission with a peak 6 h after injury, followed by a decrease during the remainder of the study period. D-dimer levels also correlated with hemorrhage progression
Nakae [19]	Retrospective	Isolated severe TBI	Adults	234	0 (0%)	Excluded those with liver failure, hematological disease or malignancy	On admission and 3, 6, 12 h after injury	< 1 h of injury	Increased D-dimer on admission with a peak 3–6 h after injury, followed by a decrease during the remainder of the study period. More severe injury was associated with earlier peak levels
Touho [35]	Prospective observational	Isolated TBI	N/A	12	N/A	N/A	On admission and 10–17 days after injury	< 24 h of injury	Increased FDP on admission as compared to 10–17 days after injury
Grenander [21]	RCT	Isolated TBI	Adults	28	0 (0%)	Excluded those with known bleeding or coagulation disorders	On admission and 8, 16, 24, 36, 48, 72, 96, 120 h after injury	Mean 8.6 h after injury	Increased D-dimer on admission, followed by a decrease during the remainder of the study period
Nekludov [41]	Prospective observational	Isolated severe TBI	Adults	11	0 (0%)	Excluded those with known coagulation disorders or alcohol abuse	On admission and 3 days after injury	15.4 ± 2.6 h after injury	Increased D-dimer on admission, followed by a decrease to a moderately elevated plateau during the following days
Gando [43]	Prospective observational	Isolated TBI	Adults	5	0 (0%)	Excluded those with preexisting coagulation disorders	On admission and 1, 2, 3, 4 days after injury	N/A	Increased D-dimer on admission, followed by a decrease during the remainder of the study period
Pahatouridis [40]	Prospective observational	Isolated moderate TBI	Adults	61	0 (0%)	Excluded those with known coagulopathy	On admission and after 4, 6, 24, 48, 72 h after injury	N/A	Increased D-dimer on admission that remained during day 1, followed by a decrease during the remainder of the study period. Lower GCS corresponded to increased D-dimer levels
DeFazio [44]	Retrospective	Isolated severe TBI	Adults	44	N/A	N/A	On admission and 24, 48, 72 h after injury	< 3 h of injury	Increased D-dimer on admission, followed by a decrease during the remainder of the study period. Median values at 24 h greater in patient with poorer clinical status
Foaud [45]	Prospective case control	Isolated TBI, admitted to ICU	Pediatric	46	N/A	N/A	On admission and day 3, 14 after injury	N/A	Increased D-dimer on admission, followed by a decrease during the remainder of the study period
Suehiro [46]	Retrospective	Moderate to severe TBI	All ages	9	N/A	N/A	On admission and 1, 3, 5, 7 days after injury	N/A	Increased D-dimer on admission, followed by a decrease during the remainder of the study period
Karri [47]	Retrospective	Severe TBI	Adults	25	N/A	N/A	On admission and 2, 4, 6 h after admission	N/A	Increased D-dimer on admission, followed by a decrease during the remainder of the study period
Scherer [34]	Prospective observational	Isolated severe TBI	Adults	20	N/A	N/A	A and 3 h after admission	N/A	Increased D-dimer highest on admission that had decreased three hours later
Hulka [27]	Retrospective	TBI	Adults	49	N/A	N/A	N/A	N/A	Immediate and persistent elevation of D-dimer concentration in 70% of the population on admission, that decreased to 40% 18 h after injury

*FDP* fibrin and fibrinogen degradation product; *h* hours; *TBI* traumatic brain injury

#### PAI-1 and t-PA

We included 4 studies with PAI-1 and/or t-PA data. In the study of 141 TBI patients by Fair et al., PAI-1 and t-PA were elevated on admission and then decreased over time [23]. A similar pattern of posttraumatic increase in PAI-1 and t-PA, with the highest levels seen on admission, was observed in the other three smaller studies as well [43, 46, 48] (Table 6).

**Table 6:** Plasminogen activators and inhibitors

Reference	Study design	TBI type	Age category	Patients (n)	Antithrombotic treatment (n, %)	Hemostatic comorbidities	Sampling times	First sample	Results
Fair [31]	Prospective observational	Isolated TBI	Adults	141	20 (14%) ASA	Excluded those with known coagulation disorders	On admission and 6, 12, 24, 48 h after injury	N/A	Increased PAI-1 and t-PA on admission that then decreased over time studied
Gando [43]	Prospective observational	Isolated TBI	Adults	5	0 (0%)	Excluded those with preexisting coagulation disorders	On admission and 1, 2, 3, 4 days after injury	N/A	Increased PAI-1 on admission that then decreased to normal levels 5 days after injury
Becker [48]	Prospective observational	Severe TBI	Pediatric	27	0 (0%)	N/A	On admission and 1, 3–5, 7–9, 21 and 35 days later	< 2 h of injury	Increased PAI-1 and t-PA on admission that then decreased over time studied
Karri [47]	Retrospective	Severe TBI	Adults	25	N/A	N/A	On admission and 2, 4, 6 h after admission	N/A	Increased t-PA on admission that then decreased over time studied. Increased PAI-1 with peak levels 4 h after admission. uPA increase over time studied

*H* hours; *PAI-1* plasminogen activator inhibitor 1; *TBI* traumatic brain injury; *t-PA* tissue plasminogen activator; *uPA* urokinase plasminogen activator

### Viscoelastic Assays

We included 5 studies with viscoelastic assay data. In a study of 91 pediatric patients with severe TBI, elevated TEG clot lysis (LY30) was noted within the first hour and decreased over time [49]. In another study by the same group, where early sampling was not performed, no significant change in TEG LY30 was detected. Similarly, in samples taken regularly during the first two days after trauma, Fair et al. found no significant change in TEG LY30, although it is unclear when the first sample was taken [23]. Another ROTEM study of 83 adults with isolated mild TBI, where blood sampling was performed 12–72 h after injury, found no significant change in clotting time (CT), clot formation time (CFT) or maximum clot firmness (MCF). Following the initial phase, two pediatric studies from the same center observed that TEG LY30 then decreased below reference ranges, indicative of fibrinolysis shutdown. In the first study, where sampling was performed on admission and once daily, fibrinolysis shutdown was the most common phenotype between postinjury day 1 and continuing through postinjury day 3 [50]. In the follow-up study, in which early sampling was performed with a higher frequency, fibrinolysis shutdown was the most dominant phenotype already 3 h after trauma [49] (Table 7).

**Table 7:** Viscoelastic assays

Reference	Study design	TBI type	Age category	Patients (n)	Antithrombotic treatment (n, %)	Hemostatic comorbidities	Sampling times	First sample	Results
Fair [31]	Prospective observational	Isolated TBI	Adults	141	20 (14%) ASA	Excluded those with known coagulation disorders	On admission and 6, 12, 24, 48 h after injury	N/A	Normal TEG LY30 during time studied
Wang [33]	Interventional	Isolated moderate TBI	Adults	83	0 (0%)	Excluded those with known coagulation disorders	NO admission, 12, 24, 48, 72 h after injury	N/A	Normal ROTEM CT, CFT or MCF during time studied
Leeper [50]	Prospective observational	Severe TBI	Pediatric	39	N/A	Excluded those with preexisting coagulation disorders	On admission and 1, 2, 3, 4 days after injury	N/A	Normal LY30 on admission that decreased to fibrinolysis shutdown ranges starting on postinjury day 1 and continuing to postinjury day 3
Leeper [49]	Prospective observational	Severe TBI	Pediatric	91	N/A	Excluded those with preexisting coagulation disorders	N/A	N/A	Elevated TEG LY30 within the first hour after injury, followed by a decrease toward fibrinolysis shutdown which had become the predominant phenotype already 3 h after trauma
Massaro [57]	Prospective observational	Moderate to severe TBI	Adults	25	0 (0%)	Alcohol use in 68%	On admission and 1–2, 2–3, 3–4, 4–5 days after injury	Mean 18 h after injury	TEG MA, TG and G higher at late stage (> 48 h) compared to early stage (0–48 h) after injury

*CFT* clot formation time; *CT* clotting time; *h* hours; *MA* maximum amplitude; *MCF* maximum clot firmness; *G* *G*-value; *TBI* traumatic brain injury; *TEG* thromboelastography; *TG* thrombin generation; *ROTEM* rotational thromboelastometry

## Discussion

TBI is often followed by hemostatic disturbance [2, 3], which has been associated with worse clinical outcome [51]. It is important to note that hemorrhagic lesion progression primarily occurs during the first hours after injury, with one study of mild TBI reporting that lesion progression had stopped in 75% of patients within 2 h, and in 97% within 24 h, of injury [52]. This highlights the window of opportunity for the treatment of deranged hemostasis. The aim of this study was to review the literature on the time course of hemostatic disruptions following TBI. Thirty-three studies were included, contributing data on temporal changes in primary hemostasis, coagulation cascade function and fibrinolysis. To the best of our knowledge, this is the first review of its kind and contributes findings that are important for patient management and future study design.

### Primary Hemostasis

Clinically, primary hemostasis is assessed by platelet count and platelet function [53]. In this review, we observed that TBI was followed by a decrease in platelet count and function that appeared to be independent of platelet transfusions and antithrombic therapy [36, 37]. Platelet count typically stayed within reference ranges [19, 20, 24–26, 29, 30, 35] and returned to admission levels within two weeks [28, 30, 31], while platelet function decreased below reference levels about 6–48 h after injury [24, 29, 37, 38]. The reason behind this is likely multifactorial. Consumption of platelet–fibrin clot formation probably decreases platelet count early on, a decrease which is aggravated by dilution due to extensive fluid resuscitation therapy. Additional decreases in platelet count and function may be the result of consumptive depletion and exhaustion [2]. Platelet dysfunction could also be driven by endothelial injury [54], with endothelial disruption catalyzing systemic activation of platelets that renders them inert to subsequent stimulation [55], as well as factors within soluble plasma that render platelets inactive after trauma [56]. Platelets also undergo structural change following trauma, which could decrease their function, but it remains unclear whether this process occurs in isolated TBI [55].

Two studies also found that platelets became hyperactive following their initial dysfunction [29, 37]. This was supported by a TEG study that noted increasing maximum amplitude (MA) levels 7 days after TBI [57] (MA represents clot strength that is mainly dependent on platelet function). Platelet hyperactivity could explain the increased risk of thrombosis seen in TBI patients [10–18, 58], but future studies are warranted to assess its clinical significance.

**Fig. 2:**
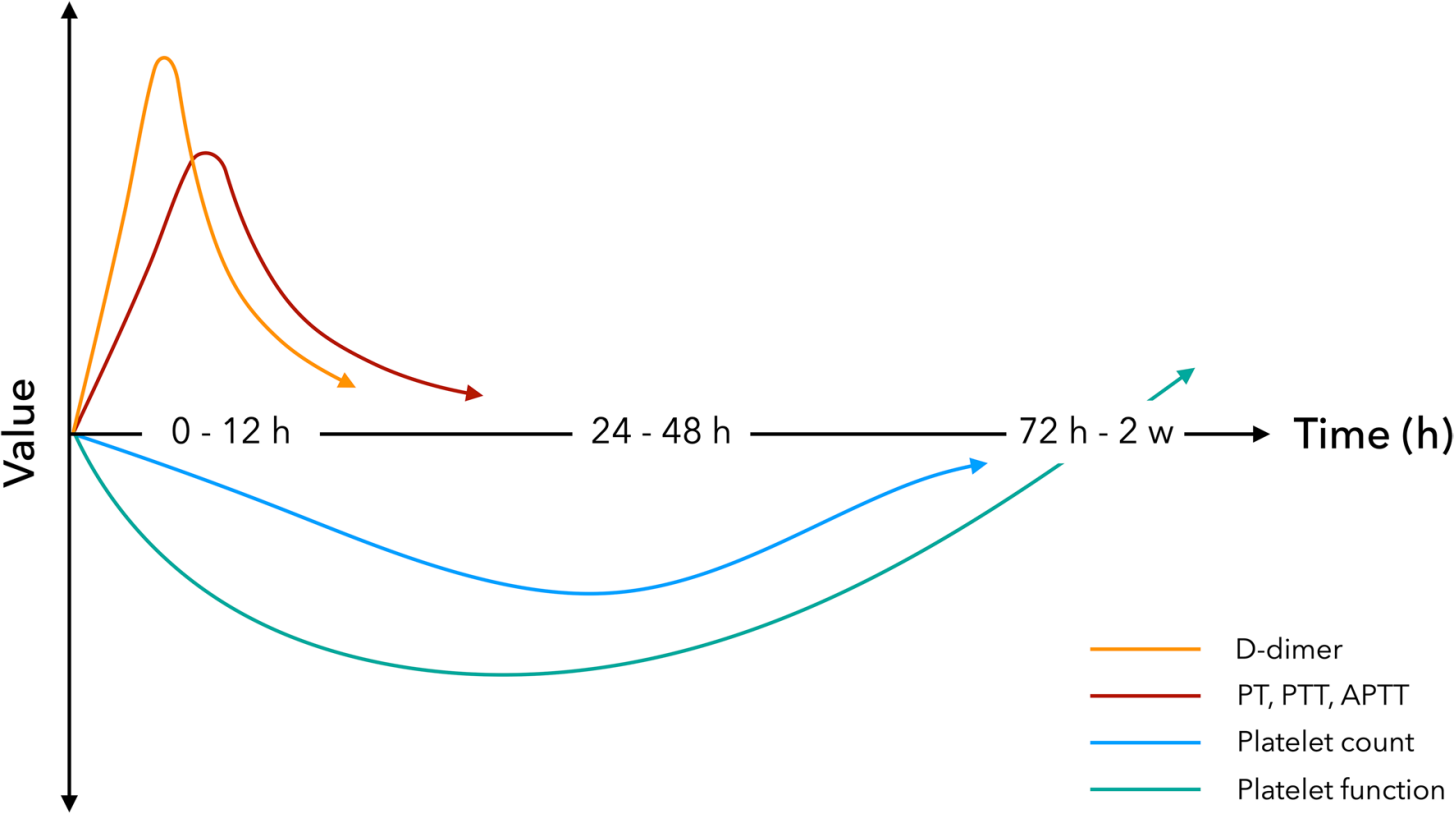
Conceptual figure of the time course of hemostatic disruptions after traumatic brain injury

In summary, platelet dysfunction and decreased platelet count were evident during the acute postinjury phase. In patients surviving the initial insult, platelets became hyperactive, although the exact time frame remains to be determined (Fig. 2).

#### Clinical Implications

Platelet dysfunction was seen between six hours to several days after injury. Considering the fact that lesion progression is most prevalent within the first hours of trauma [52], this might imply that platelet alterations are not a main cause behind exacerbation of hemorrhagic lesions in TBI. This is supported by the fact that posttraumatic platelet dysfunction has not been associated with intracranial lesion progression [37]. However, while hemorrhagic expansion of the primary brain contusions is a chief concern, delayed noncontiguous hemorrhagic lesions at remote locations may also appear in the evolution of a patient’s clinical course [59]. This could be caused by platelet dysfunction. Further adding to the uncertainty regarding the clinical significance of platelet dysfunction in TBI, platelet function assays were originally designed to detect antiplatelet medication efficacy, and have secondarily been extrapolated to trauma, making interpretation of cutoff values unclear. The assays are also limited by the absence of endothelium and flow conditions, as well as variability related to platelet count and hematocrit level [54]. Highlighting this, studies of platelet function-targeted therapy have failed to demonstrate clinical efficacy. While there is some evidence that platelet transfusion can correct platelet dysfunction in TBI [60], others studies suggest that transfusions are more likely to improve aspirin-induced, rather than trauma-induced, dysfunction [24]. Desmopressin could be an alternative to platelet transfusions, though there exist only mixed results from low-quality studies [61, 62]. Thus, it is unclear whether posttraumatic platelet dysfunction contributes to lesion progression in TBI, and there is also no proven treatment for TBI-induced platelet dysfunction.

### Coagulation Cascade

In the clinical setting, coagulation cascade function is assessed by PT, PTT and APTT, as well as surrogate markers of thrombin generation such as TAT and F1 + 2. We observed that TBI was followed by a temporary increase in APTT, PTT and PT [19, 21, 33, 39, 40], indicating decreased coagulation cascade function. This seemed to be apparent on admission and was most pronounced during the first hours after injury [19, 21, 33, 40]. TAT and F1 + 2 were also elevated during this time [26, 27, 41–43], indicating that the coagulation cascade dysfunction was secondary to increased thrombin generation. Therefore, the changes are probably the result of consumption, where excess clotting leads to depletion and/or dysfunction of coagulation factors in plasma [39]. Many believe that this is due to trauma-induced unregulated tissue factor release [58, 63], which would stimulate thrombin generation and consumption of clotting factors in the ways observed in this review, but evidence remains elusive. Thus, the first few hours following TBI are characterized by coagulation cascade dysfunction that is likely secondary to unregulated thrombin production (Fig. 2).

#### Clinical Implications

Coagulation cascade dysfunction appears to occur when the risk of intracranial lesion progression is high [52], supporting a theory that it contributes to hemorrhagic expansion in TBI. This presents a possible window of opportunity for interventions. In general trauma, there has been much interest in the use of fresh frozen plasma (FFP), which contains both fibrinogen and clotting factors [64], to limit blood loss [2]. In isolated TBI, retrospective evidence has found that early plasma transfusion is associated with improved survival in patients with multifocal intracranial hemorrhage [65], but no randomized controlled trials have been performed to validate this. Prothrombin complex concentrate (PCC) provides another theoretical treatment strategy for coagulation cascade dysfunction secondary to consumption of clotting factors, and is currently recommended for the emergency reversal of vitamin K anticoagulant therapy in TBI [2]. One retrospective study of general trauma patients found that PCC decreased mean INR in patients without warfarin treatment [66], but there is currently no recommendation for its use in patients with isolated TBI without anticoagulation therapy [67]. Administration of recombinant FVIIa has also been associated with reduced hemorrhage progression in TBI, albeit with a higher rate of thrombosis, but the effect size was small and the benefit of routine recombinant FVIIa remains unclear [68]. Thus, there is a sound theoretical basis for the belief that consumption of clotting factors contributes to lesion progression in TBI, but there are yet no evidence-based treatment options to prevent this.

### Fibrinolysis

Fibrinolysis is usually assessed using D-dimer and, to a certain extent, viscoelastic assays. Some studies also use t-PA, which stimulates conversion of plasminogen to plasmin, as well as PAI-1, an inhibitor of t-PA [69]. We observed an increase in D-dimer following TBI [19, 23, 26, 27, 33, 35, 40, 41, 43–47], with peak levels seen during the first 6 h after injury [19, 23, 40]. This shows that TBI is followed by an early increase in fibrinolytic activity, which also seems to be proportional to injury severity [19]. This is supported by the observation that PAI-1 and t-PA are increased during the same time period [23, 43, 46, 48]. However, only one study could verify this using viscoelastic assays during the first hour after injury [49]. This could mean that hyperfibrinolysis is only present within this short time interval, or be due to an arbitrary threshold for hyperfibrinolysis [70] and LY30′s relative lack of sensitivity to changes in fibrinolysis [23]. Highlighting this, a study of general trauma found that 90% of patients with hyperfibrinolysis on D-dimer, t-PA and plasmin‐α2AP did not meet criteria based on TEM, leading the authors to conclude that TEM may be an unreliable measurement of endogenous fibrinolytic activity [71]. Thus, hyperfibrinolysis is evident in the acute phase following TBI, but it remains unclear exactly for how long it continues (Fig. 2).

#### Clinical Implications

It has been suggested that hyperfibrinolysis is a main driving force behind trauma-induced bleeding disorders in TBI, and can cause hemorrhage expansion via the degradation of coagulation factors, breakdown of the formed fibrin clot, and impairment of clot formation as a result of excessive generation of fibrin degradation products. Moreover, from a time-based standpoint, hyperfibrinolysis appears to occur when the risk of intracranial lesion progression is high [52]. A subgroup analysis from the CRASH-3 randomized trial found that administration of tranexamic acid (TXA) was associated with a reduction in 28-day head injury-related mortality if administered within 3 h to patients with mild-to-moderate isolated TBI [72]. This indicates that antifibrinolytic drugs can provide effective treatment. However, the CRASH-3 study protocol was modified, reducing the time from injury to randomization from 8 to 3 h, and no treatment effect was seen in patients with severe TBI. Limiting the time window after TBI probably increased the chance that TXA was administered when hyperfibrinolysis actually took place. Based on the observations in this review, one would expect prehospital administration of TXA to be even more effective, a treatment option which has been assessed in a completed but not yet published trial (ClinicalTrials.gov Identifier: NCT01990768). Other antifibrinolytic drugs, such as aprotinin, epsilon-aminocaproic acid and aminomethylbenzoic acid, are all also theoretically sound options but have not been assessed in TBI. Thus, early hyperfibrinolysis appears to contribute to lesion progression, and there is evidence in support of antifibrinolytic treatment in a subgroup of TBI patients if administered early.

### Prothrombotic State

TBI is also independently associated with an increased risk of thromboembolic complications that persist after index hospitalization [10–18, 73]. Most likely, initial hypocoagulability transitions to a subacute hypercoagulability phase. Recent evidence suggests that posttraumatic fibrinolytic shutdown is associated with a higher risk of thromboembolic events in TBI [74]. In the pediatric study by Leeper et al., fibrinolysis shutdown (as indicated by low TEG LY30 levels) was the predominant phenotype already 3 h after injury [49]. Similarly, in their study using TEM in adult patients with moderate-severe TBI, Massaro et al. identified a progressive and delayed hypercoagulable state that was more common > 48 h, compared to < 48 h, after injury [57]. With regard to platelet function, two studies found that multiplate levels increased above reference ranges 1–2 weeks following initial trauma [29, 37]. Collectively, these results indicate a delayed prothrombotic state characterized by initial fibrinolysis shutdown, which appears within hours to days of injury, and later possible platelet hyperactivity. However, the time point at which a patient becomes phenotypically prothrombotic remains unclear, and will most likely be a combination of these factors countered by any residual coagulation cascade dysfunction and thrombocytopenia. These findings warrant particular consideration with regard to several unresolved clinical dilemmas in order to decrease the rates of VTEs and capillary microthrombi seen in vulnerable TBI patients, such as the timing of postinjury pharmacological thromboprophylaxis and resumption of oral anticoagulation therapy. However, more studies on long-term hemostatic disruptions after TBI are needed before any further conclusions can be drawn.

### Age Differences

The three studies that included patients of all ages did not perform any analysis to differentiate between adult and pediatric populations [32, 42, 45]. In the four studies that focused exclusively on pediatric populations, posttraumatic elevations in D-dimer [44], PAI-1, t-PA [48] and TEG LY30 [49, 50] were noted. Thus, pediatric patients seem to experience posttraumatic hyperfibrinolysis, but a direct comparison to adults is lacking. Moreover, to date, no studies have assessed whether pediatric patients undergo posttraumatic changes in platelet count, platelet function and coagulation cascade function as well.

### Systemic Versus Localized Hemostasis

The primary contusion in TBI is the result of ruptured microvessels. In the penumbra and surrounding regions, molecular processes can then be activated leading to hemorrhagic progression or new lesions [75]. The addition of hemostatic disturbance contributes to lesion progression [76]. For all studies included in this review, the assumption has been made that hemostatic assays sampled from the systemic circulation are applicable to the brain vasculature as well. However, the complexity of the cerebral microvessel walls, and their differences in structure and function compared to extracranial vessels, might suggest that hemostatic response differs regionally [77]. In severe sepsis for example, certain organs have been found to be at a greater risk of thrombosis than others, indicating that hemostatic disruption might partly be a localized phenomenon [78]. With this said, there is still very little evidence that blood or plasma is altered in its passage through the brain, or that the brain environment alters blood components under normal conditions [79].

### Study Limitations

There are some limitations to this study that should be mentioned. For one, TBI is heterogeneous and multifactorial, consisting of a mix of pathologic processes that can be active in different patients and in different regions of the brain. In addition, the methodological quality of the included studies reviews was variable, with many being observational cohort studies without control groups and variable numbers of participants. Studies also varied in terms of sampling frequency and follow-up time. Different cutoff levels and parameters were also used for the hemostatic assays, which makes standardization and interpretation difficult. We also only made use of a single search database to perform our systematic review, which could exclude potentially valuable articles [80]. However, we also screened the reference lists of the included studies and thus consider this risk to be minor. Despite its limitations, we believe that clinically helpful and valid conclusions can be drawn from this review, and will be useful in designing future studies and clinical practice guidelines.

## Conclusions and Future Directions

Altered hemostasis, with early hemorrhagic progression and later thrombotic complications, is a substantial and ongoing challenge in the management of TBI. There is currently a lack of well-designed prospective trials reviewing the temporal trend in hemostatic disruption, making it difficult to identify windows of opportunity for potential treatment options. Moreover, most data underlying the initiation and progression of hemostatic disruption in TBI are speculative and not linked to causative data. Based on the results from this review, it appears that TBI is followed by both early and delayed hemostatic disturbance. The first hours after TBI are characterized by a derangement of the coagulation system, as a consequence of massive and systemic activation, and hyperfibrinolysis, both of which likely contribute to lesion progression. This is then followed by decreased platelet function and count, the clinical significance of which remains unclear. Thereafter, a poorly defined prothrombotic state emerges, which might be characterized by fibrinolysis shutdown and hyperactive platelets. In the clinical setting, only early administration of TXA has proved effective in reducing the risk of head-injury related mortality in a subgroup of TBI patients. Further studies aimed at analyzing the progress of hemostatic disruption following TBI are warranted. In particular, studies with high-frequency early sampling are warranted in order to characterize the cause behind intracranial lesion progression, as well as those with longer follow-up times in order to understand the delayed prothrombotic state. Studies using point-of-care viscoelastic assays are also of particular interest, as they have the potential of providing real-time information in the clinical setting.

## Electronic supplementary material

Below is the link to the electronic supplementary material.

Supplementary file1 (DOCX 15 kb)

## Abbreviations

AA: Arachidonic acid
ADP: Adenosine diphosphate
AIS: Abbreviated injury scale
APTT: Activated partial thromboplastin time
ASPI: Aspirin
CFT: Clotting formation time
COL: Collagen
CT: Clotting time
F1 + 2: Prothrombin fragment 1 + 2
FFP: Fresh-frozen plasma
GCS: Glasgow coma scale
LY30: Lysis index 30
MA: Maximum amplitude
MCF: Maximum clot firmness
PAI-1: Plasminogen activator inhibitor-1
PCC: Prothrombin complex concentrate
PT: Prothrombin time
PTT: Partial thromboplastin time
t-PA: Tissue plasminogen activator
TAT: Thrombin antithrombin III complex
TBI: Traumatic brain injury
TEG: Thromboelastography
TEM: Thromboelastometry
TRAP: Thrombin receptor-activating peptide
TXA: Tranexamic acid
