# Supplementary material for: Time Course of Hemostatic Disruptions After Traumatic Brain Injury: A Systematic Review of the Literature
Source: Neurocrit Care. 2020 Jun 30;34(2):635–56. doi: 10.1007/s12028-020-01037-8 (PMC8128788; doi:10.1007/s12028-020-01037-8)
Supplement: Supplementary file 1 — Supplementary file1 (DOCX 15 kb) [file 12028_2020_1037_MOESM1_ESM.docx]

**MEDLINE search Strategy**

1. traumatic brain injury
2. brain injury
3. head injury
4. head trauma
5. brain trauma
6. craniocerebral trauma
7. 1 or 2 or 3 or 4 or 5 or 6
8. coagulopathy
9. coagulation
10. hemostasis
11. thromboplastin time
12. prothrombin time
13. prothrombin ratio
14. international normalized ratio
15. prothrombin fragment
16. thrombin–antithrombin complex
17. thromboelastography
18. thromboelastometry
19. ROTEM
20. thrombocytopenia
21. platelet count
22. platelet function
23. platelet dysfunction
24. platelet aggregability
25. multiplate
26. verifynow
27. fibrinolysis
28. fibrinogen
29. d-dimer
30. 8 or 9 or 10 or 11 or 12 or 13 or 14 or 15 or 16 or 17 or 18 or 19 or 20 or 21 or 22 or 23 or 24 or 25 or 26 or 27 or 28 or 29 or 30

**Search string was “7 and 30”**
